# Supplementary material for: Bioengineered textiles with peptide binders that capture SARS-CoV-2 viral particles
Source: Commun Mater. 2022 Aug 15;3(1):54. doi: 10.1038/s43246-022-00278-8 (PMC9376897; doi:10.1038/s43246-022-00278-8)
Supplement: Supplementary file 1 — Description of Additional Supplementary Files [file 43246_2022_278_MOESM1_ESM.pdf]

Description of Additional Supplementary Files

File Name: Supplementary Data 1

Description: Plasmid list, complete construct sequences and raw data for all figures including statistical analysis.
