# Supplementary material for: Bioengineered textiles with peptide binders that capture SARS-CoV-2 viral particles
Source: Commun Mater. 2022 Aug 15;3(1):54. doi: 10.1038/s43246-022-00278-8 (PMC9376897; doi:10.1038/s43246-022-00278-8)
Supplement: Supplementary file 2 — Supplementary Information [file 43246_2022_278_MOESM2_ESM.pdf]

Supplementary Information for 'Bioengineered textiles with peptide binders that capture SARS-CoV-2 viral particles'.

Laura Navone[1,2,*], Kaylee Moffitt[1], Wayne A. Johnston[1,2], Tim Mercer[3,4], Crystal Cooper[5], Kirsten Spann[6] and Robert E. Speight[1,2]

[1] School of Biology and Environmental Sciences, Faculty of Science, Queensland University of Technology (QUT), Brisbane, Queensland, 4000, Australia.

[2] ARC Centre of Excellence in Synthetic Biology, Queensland University of Technology (QUT), Brisbane, Queensland, 4000, Australia.

[3] Australian Institute for Bioengineering and Nanotechnology, The University of Queensland (UQ), Brisbane, Queensland, 4072, Australia.

[4] Garvan Institute of Medical Research, Sydney, NSW 2010, Australia.

[5] Central Analytical Research Facility (CARF), Queensland University of Technology (QUT), Brisbane, Queensland, 4000, Australia.

[6] Centre for Immunology and Infection Control, School of Biomedical Science, Faculty of Health, Queensland University of Technology (QUT), Brisbane, Queensland, 4000, Australia.

*Corresponding author: laura.navone@qut.edu.au, Queensland University of Technology, Brisbane, Queensland, Australia.

Production of capture peptides and selection of transformants.

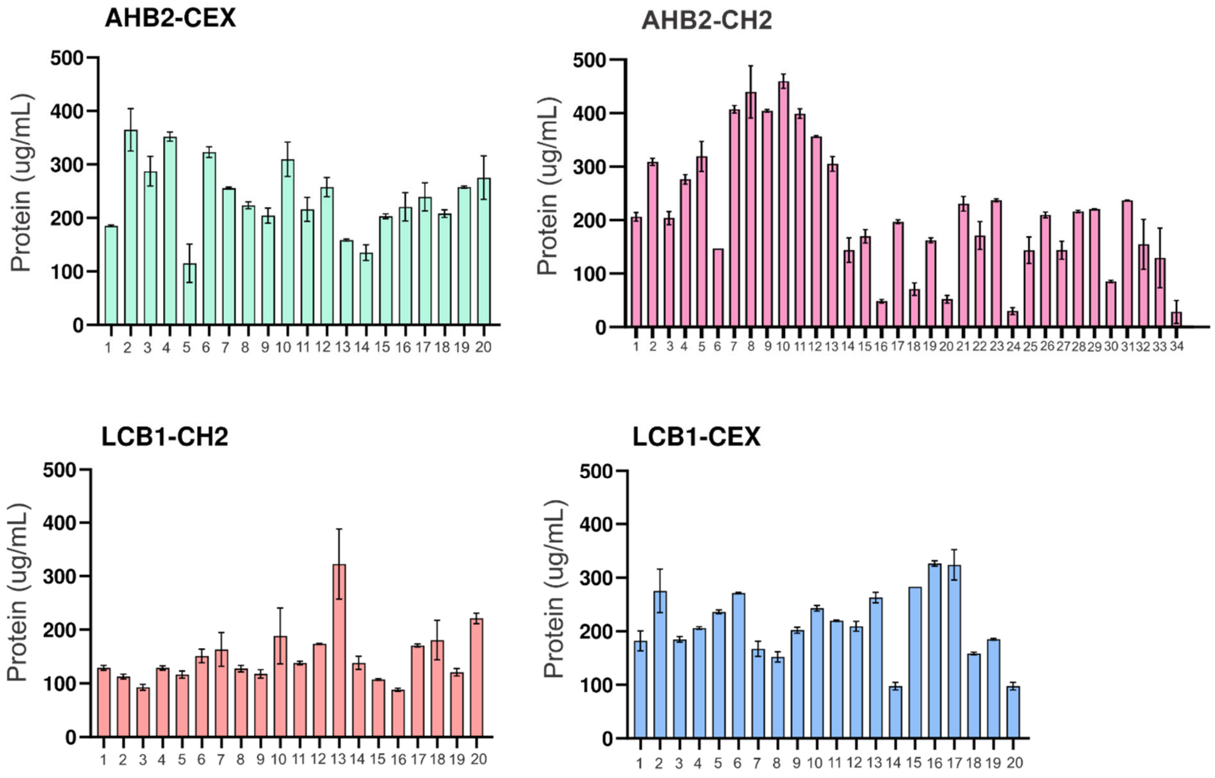

(over) Supplementary Fig. 1. Manufacture of capture peptides in *Pichia pastoris*. We used *P. pastoris* as production platform due to its a high secretory capability for heterologous proteins and low production costs which are suitable for manufacture at scale. Engineering of *P. pastoris* strains to achieve high productivity requires extensive optimisation, and extensive screening and selection of best producer clones due to genetic variability during genome integration events. Each column in bar graphs indicate capture peptide production by different *P. pastoris* transformants in flask fermentations measured by protein concentration in the supernatant.

a. **Langmuir Isotherms of capture peptides absorption to cellulose.**

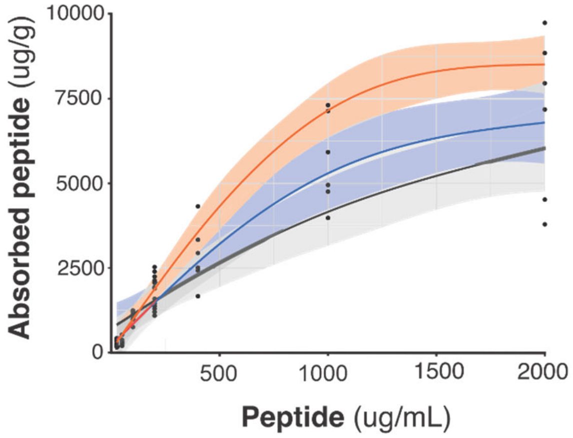

b. **Binding parameters of capture peptide to cellulose.**

	**Bmax** (umol/g)	**Kd**(pM)
**Rayon**	0.71 ± 0.1	60.7 ± 23.8
**Cotton**	0.91 ± 0.1	61.3 ± 14.4
**Avicel**	0.52 ± 0.1	92.0 ± 38.2

C. **Binding of capture peptides to cellulose at different pH.**

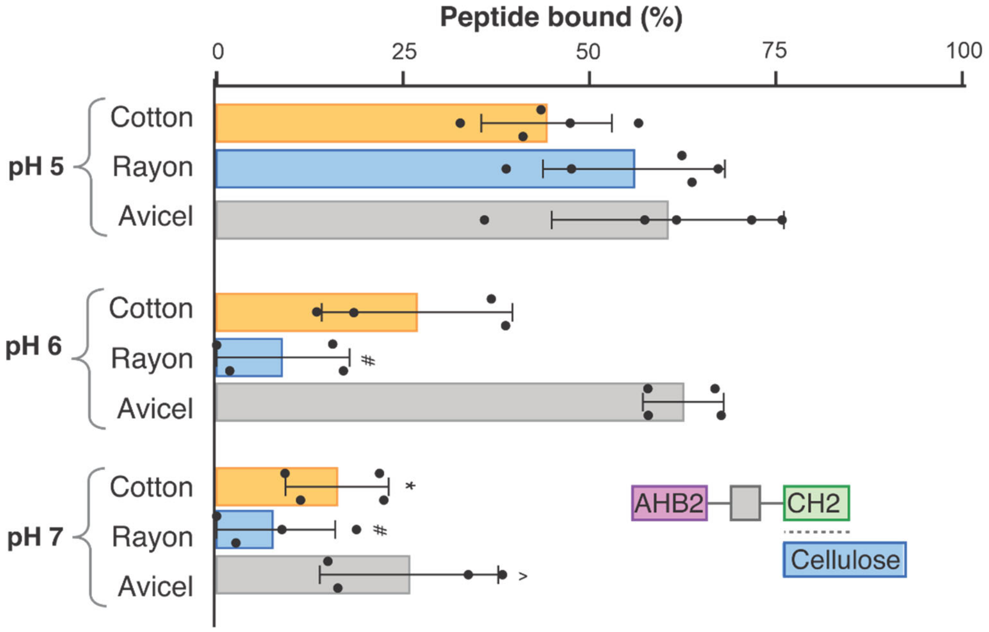

d. Binding of capture peptides to cellulose at 4 °C.

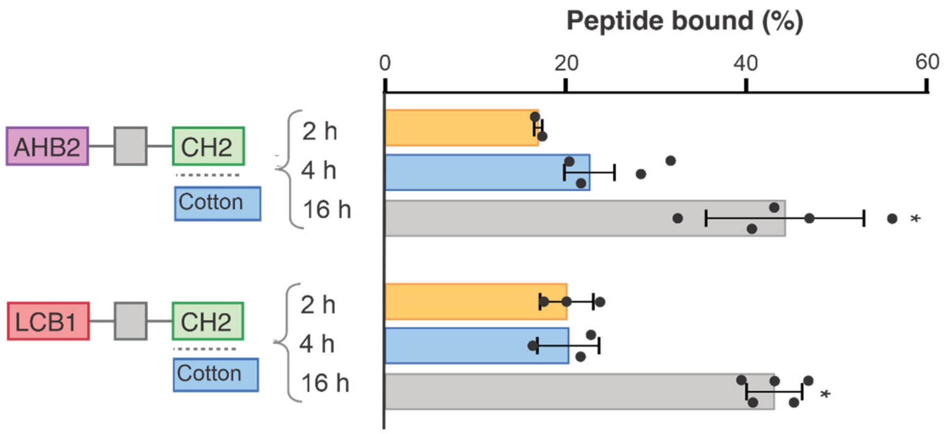

e. Binding of capture peptides to cellulose at room temperature.

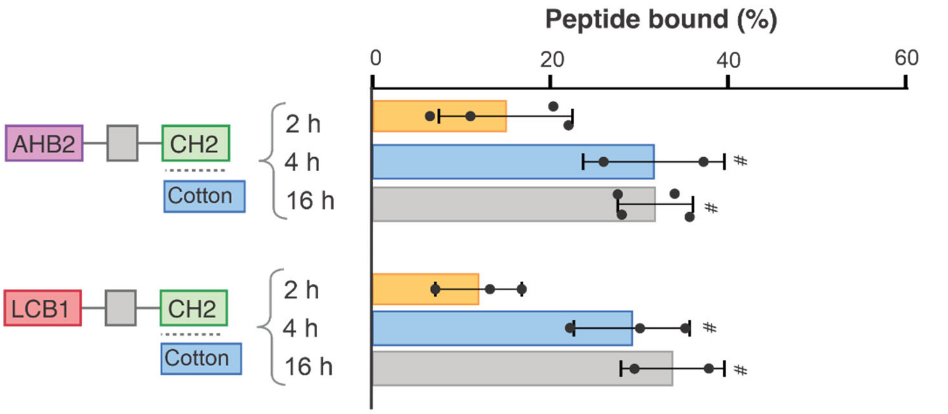

f. Washing and removal of capture peptides.

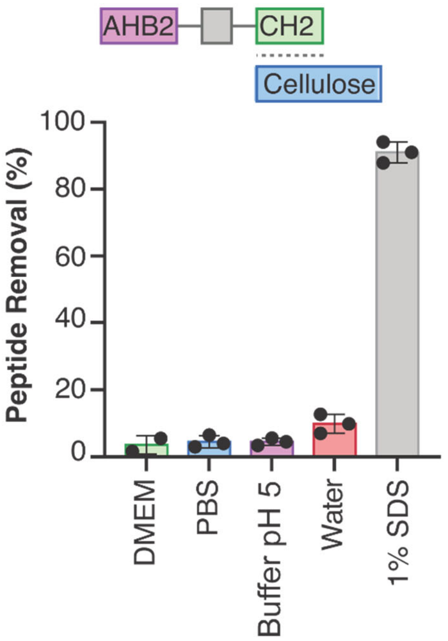

30

(over) Supplementary Fig. 2. Binding capability of capture peptides to cellulose. We performed isothermal absorption assays to analyse the binding affinity of the capture peptides to cellulose. **a,** Langmuir isotherms of capture peptide AHB2-CH2 for cotton, rayon and Avicel. Solid lines represent the mean of the model fits, full black circles represent raw data and shading is the standard error of model fits. The fit of the Langmuir model showed a highly significant effect to the data when pooled (Adj. R2 = 0.90; $p < 0.001$). For each substrate type, model fits were highly significant for Avicel (Adj. $R^2 = 0.79$; $p < 0.001$), cotton (Adj. $R^2 = 0.87$; $p < 0.001$), and rayon (Adj. $R^2 = 0.95$; $p < 0.001$). **b,** Bmax and K_d for of AHB2-CH2 for cotton, rayon and Avicel calculated from the Langmuir isotherm model fit ($p < 0.001$). We also measured the binding of AHB2-CH2 to cotton, rayon and Avicel to test the binding at pH 6 and 7, compared to pH 5. **c,** Binding of capture peptide AHB2-CH2 binding to cotton, rayon or Avicel at pH 5, 6 and 7. Data are presented as mean and standard deviation of biological triplicates and analysed using one-way ANOVA with multiple comparisons. Individual data points are shown by filled black circles. *Significantly different to AHB2-CH2 binding to cotton at pH 5 ($p < 0.01$). #Significantly different to AHB2-CH2 binding to rayon at pH 5 ($p < 0.01$) ^Significantly different to AHB2-CH2 binding to Avicel at pH 5 ($p < 0.01$). We next evaluated whether the bioengineered cotton retained the capture-peptides during manufacture and washing conditions. **d,** Binding of capture peptide AHB2-CH2 to cotton for two, four or sixteen hours at 4°C or **e,** at room temperature. Data are presented as mean and standard deviation of biological triplicates and analysed using one-way ANOVA with multiple comparisons. Individual data points are shown by filled black circles. *Significantly different to AHB2-CH2 binding to cotton for 2 and 4 hours at 4°C ($p < 0.01$). #Significantly different to AHB2-CH2 binding to cotton at 2 hours at room temperature ($p < 0.01$). **f,** Washing and removal of capture peptide with DMEM, PBS, phosphate buffer pH 5, deionised water or 1% SDS.

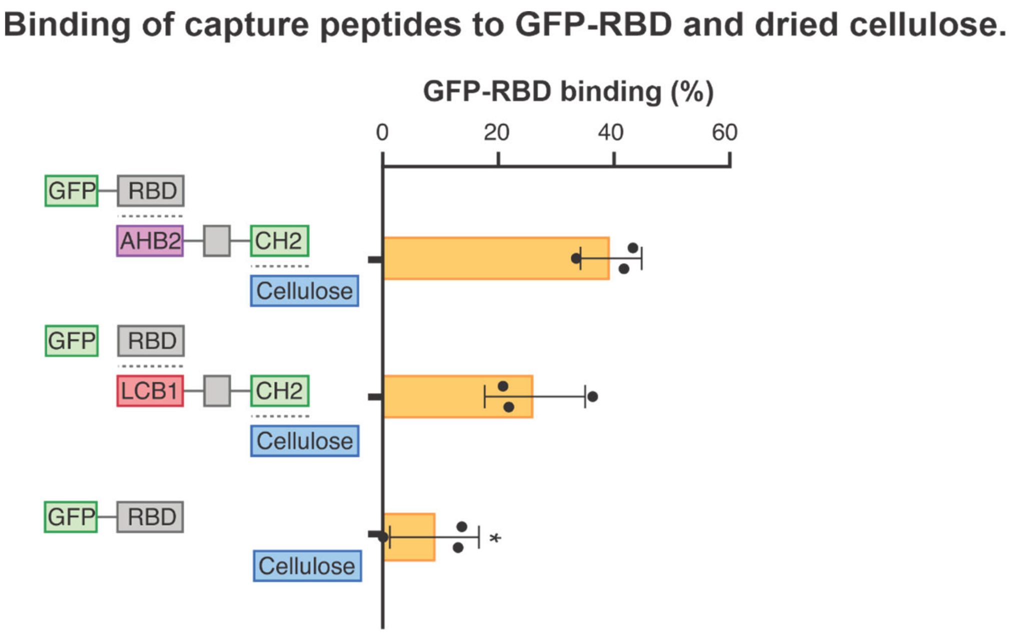

Supplementary Fig. 3. Binding of GFP-(SARS-CoV-2 RBD) to dried bioengineered textiles. We evaluated the capturing capability of dry bioengineered textiles for SARS-CoV-2 RBD under conditions similar to when respiratory droplets with viral particles might encounter dry textiles. We performed a binding experiment with GFP-RBD reporter using dried bioengineered cotton. Percentage of GFP-(SARS-CoV-2 RBD) binding to bioengineered cotton or unbound cotton is shown. Data are presented as the mean and standard deviation of biological replicates and analysed using one-way ANOVA with multiple comparisons. Individual data points are shown by filled black circles. *Significantly different to GFP-RBD/AHB2-CH2 binding ($p < 0.001$).

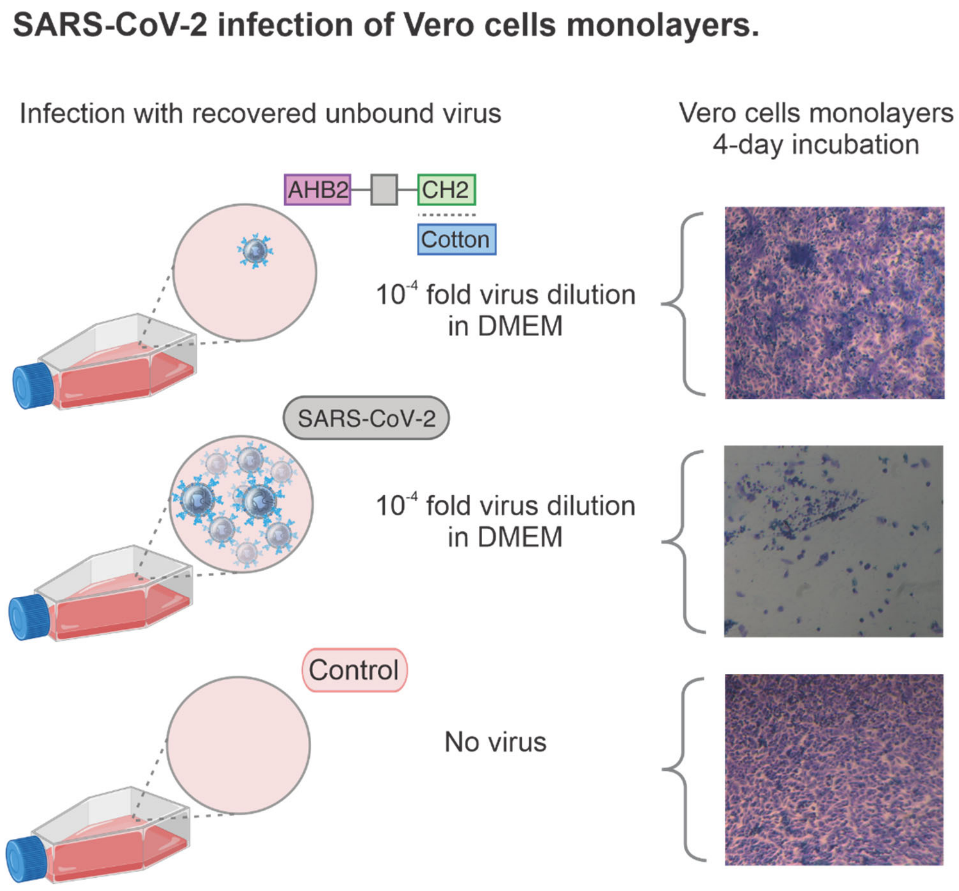

Supplementary Fig. 4. SARS-CoV-2 infection of Vero cells monolayers. We investigated the protective capability of the bioengineered cotton to SARS-CoV-2 infection. The sequestration of the viral particles reduced onward infection of Vero cell cultures. Optical microscopy photographs of Vero cell monolayers stained with Crystal Violet after infection with recovered unbound SARS-CoV-2 are shown. Control photograph shows not infected Vero cells monolayers.
